# Hippocampus-Dependent Goal Localization by Head-Fixed Mice in Virtual Reality

**DOI:** 10.1523/ENEURO.0369-16.2017

**Published:** 2017-05-02

**Authors:** Masaaki Sato, Masako Kawano, Kotaro Mizuta, Tanvir Islam, Min Goo Lee, Yasunori Hayashi

**Affiliations:** 1 RIKEN Brain Science Institute, Wako, Saitama 351-0198, Japan; 2 PRESTO, Japan Science and Technology Agency (JST), Kawaguchi, Saitama 332-0012, Japan; 3 Department of Pharmacology, Kyoto University Graduate School of Medicine, Kyoto 606-8501, Japan; 4 Department of Pharmacology, Pharmacogenomic Research Center for Membrane Transporters, Yonsei University College of Medicine, Seoul 120-752, Republic of Korea; 5 Brain and Body System Science Institute, Saitama University, Saitama, 338-8570, Japan; 6 School of Life Science, South China Normal University, Guangzhou 510631, China

**Keywords:** autism, hippocampus, mice, Shank, spatial memory, virtual reality

## Abstract

The demonstration of the ability of rodents to navigate in virtual reality (VR) has made it an important behavioral paradigm for studying spatially modulated neuronal activity in these animals. However, their behavior in such simulated environments remains poorly understood. Here, we show that encoding and retrieval of goal location memory in mice head-fixed in VR depends on the postsynaptic scaffolding protein Shank2 and the dorsal hippocampus. In our newly developed virtual cued goal location task, a head-fixed mouse moves from one end of a virtual linear track to seek rewards given at a target location along the track. The mouse needs to visually recognize the target location and stay there for a short period of time to receive the reward. Transient pharmacological blockade of fast glutamatergic synaptic transmission in the dorsal hippocampus dramatically and reversibly impaired performance of this task. Encoding and updating of virtual cued goal location memory was impaired in mice deficient in the postsynaptic scaffolding protein Shank2, a mouse model of autism that exhibits impaired spatial learning in a real environment. These results highlight the crucial roles of the dorsal hippocampus and postsynaptic protein complexes in spatial learning and navigation in VR.

## Significance Statement

We show that transient pharmacological blockade of fast glutamatergic synaptic transmission in the dorsal hippocampus in mice dramatically and reversibly impaired the retrieval of cued goal location memory acquired in a head-fixed virtual environment. Encoding and updating of virtual cued goal location memory was impaired in Shank2-deficient mice that mimic a deletion found in human autistic patients and exhibit impaired spatial learning in a real environment. These results highlight the crucial roles of the dorsal hippocampus and postsynaptic protein complexes in spatial learning and navigation in virtual reality (VR) and demonstrate the potential broad applicability of our new behavioral paradigm for molecular genetic and neural circuit studies of learning and memory as well as brain disorders.

## Introduction

A critical step in successful goal-directed navigation is the correct recognition of places. The hippocampus plays a fundamental role in spatial navigation and memory, as evidenced most strikingly by the location-specific activity of hippocampal pyramidal neurons (O’Keefe and Dostrovsky, 1971). These “place cell” properties are thought to be shaped primarily by visual and other sensory inputs from environmental cues and self-motion cues, such as proprioception and vestibular signals (Moser et al., 2008). The hippocampus in rodents receives spatial information via the postrhinal cortex and the medial entorhinal cortex, whereas nonspatial information is received through the perirhinal cortex and the lateral entorhinal cortex (Knierim et al., 2006; Manns and Eichenbaum, 2006). Thus, the hippocampus combines these two streams of information to create a “cognitive map” (Tolman, 1948; O’Keefe and Nadel, 1978), which is thought to underlie not only spatial navigation but also object–location associations and the memory for events, called episodic memory (Knierim et al., 2006; Manns and Eichenbaum, 2006).

Virtual reality (VR), which creates the perception of reality in a simulated environment, has emerged as an important tool in rodent neuroscience since the first report that rats can navigate virtual environments (Hölscher et al., 2005; Dombeck and Reiser, 2012). The range of virtual environments used for head-fixed mice in hippocampal research has now expanded from prototypical visual-based environments (Harvey et al., 2009; Dombeck et al., 2010) to tactile-based (Royer et al., 2012) and multimodal environments (Rajasethupathy et al., 2015; Danielson et al., 2016). These different environments have been used to study intracellular dynamics (Harvey et al., 2009), local circuit mechanisms (Royer et al., 2012), cellular maps (Dombeck et al., 2010) and dendritic activity (Sheffield and Dombeck, 2015) of place cells using a variety of state-of-the-art techniques, including whole-cell recordings, multichannel extracellular recordings and calcium imaging in behaving animals. However, there is limited evidence to indicate that head-fixed mice indeed remember and recognize particular virtual locations by the use of information stored in the hippocampus and that spatial learning in virtual and real environments shares common molecular genetic mechanisms.

To elucidate the neural and molecular mechanisms underlying spatial navigation and memory in VR, we developed a novel cued goal location task in VR for head-fixed mice. In this task, mice start running from one end of a virtual linear track with local and global cues to seek water rewards given at a target zone in the middle of the track. The mice need to visually locate the target zone and stay there for a short period of time to be rewarded. We show that microinfusion of the AMPA/kainate-type glutamate receptor antagonist 6-cyano-7-nitroquinoxaline-2,3-dione (CNQX) to the dorsal hippocampus dramatically and reversibly impaired learned performance of the task. Furthermore, mice deficient in the postsynaptic scaffolding protein Shank2, a mouse model of autism spectrum disorder that exhibits impaired spatial learning in the Morris water maze (Won et al., 2012), also exhibited impaired learning and relearning of cued goal localization in VR. These findings demonstrate that the encoding and updating of goal location memory in VR occurs via a postsynaptic protein complex-mediated mechanism common to spatial learning in the real world and that transient inactivation of hippocampal excitatory synaptic activity prevents retrieval of the previously acquired memory for virtual goal locations but does not permanently abolish its stored memory traces.

## Materials and Methods

### VR setup

**Figure 1.**
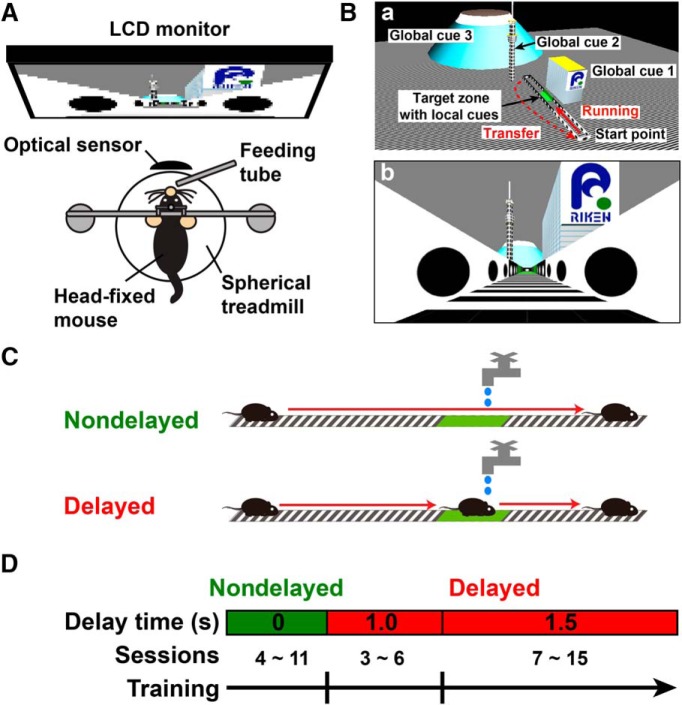
VR setup, task design, and training schedule. ***A***, The VR setup used in this study. A mouse was head-fixed above a spherical treadmill, and the rotation was detected by an optical motion sensor. The signal was fed to a program that renders VR scenes as visual feedback on a wide LCD monitor placed in front of the mouse. A water reward was delivered through a feeding tube put in front of the mouse’s mouth. ***Ba***, A bird’s-eye view of the virtual linear track with local and global visual cues. A mouse starts running along the virtual linear track unidirectionally from one end (start point) to the other. On arrival at the far end, the mouse’s position is set back to the start position (transfer). The target zone defined in the middle is denoted with green panels on the walls and floor (target zone with local cues). Large objects put outside the track serve as global cues. ***Bb***, A camera view of the virtual linear track. ***C***, Schematic representation of two different versions of the task. In the nondelayed task, mice are rewarded immediately whenever they enter the target zone. In the delayed task, mice need to stay in the target zone for a period of 1.0–1.5 s to receive the reward. ***D***, The training schedule. Training starts with several sessions of the nondelayed task, followed by the delayed task with increasing periods of time from 1.0–1.5 s.

The VR setup for head-fixed mice was built and used similarly to that described previously (Fig. 1*A*; Harvey et al., 2009; Youngstrom and Strowbridge, 2012). The system consists of a 20-cm in diameter Styrofoam spherical ball floating on air in a hemispherical metal enclosure. A mouse can be placed on the apex of the ball and head-fixed via a metal head plate. A single wide-screen 23-inch LCD display (Dell U2312) placed 30 cm in front of the subject presents a VR scene rendered by OmegaSpace 3.1 (Solidray Co Ltd) running on a Windows 7 computer in 81° horizontal and 51° vertical fields of view. The LCD monitor is sufficiently large to cover the majority of the mouse’s binocular and monocular visual fields (Sato and Stryker, 2008). Rotations of the ball were detected with a USB optical computer mouse (Logitech G400) via custom drivers and software in LabVIEW (National Instruments). The rotational velocity along the horizontal axis (aligned parallel to the mouse’s sagittal plane) generated by the head-fixed mouse running forward was converted into analog voltages. These voltage signals were then used as input signals to move the mouse’s position in the virtual environment in OmegaSpace via a USB joystick controller (BU0836X, Leo Bodner).

A water-feeding tube connected to a valve (O’Hara & Co Ltd) was positioned in front of the mouse’s mouth to deliver a water reward. The valve was triggered by the OmegaSpace script when an event to be rewarded occurred in the virtual environment. The mouse’s location in the virtual environment, the control signals to the valve for water rewards and the rotational velocity signals of the spherical treadmill were recorded using custom software in LabVIEW.

### Mice and surgery

All animal experiments were performed in accordance with RIKEN Animal Experiments Committee’s regulations.

Male C57BL/6J mice, and male and female Shank2-deficient mice, along with their wild-type littermates (Won et al., 2012, RRID:MGI:5437480), were used in this study (23–30 g body weight at the time of surgery, 11–18 weeks old at the beginning of behavioral training). In experiments using Shank2-deficient mice, data were compared between groups of homozygous mutant and wild-type littermate mice (three males and three females for mutant and three males and four females for wild-type mice). Stainless steel head plates for head fixation were surgically attached to the skull as follows. Mice were anesthetized with isoflurane (3% induction, 1.5% maintenance) and a circular piece of scalp was removed. Custom stainless steel head plates (25 mm length, 4 mm width, and 1 mm thickness) with a central opening (10 mm outer diameter and 7 mm inner diameter) were cemented to the skull using dental acrylic that covered the entire surface of the skull and anchor screws on the skull. The mice were allowed to recover from anesthesia and then returned to their home cages.

### Behavior

One week after the surgery, the mice implanted with the head plates were acclimated to handling and the Styrofoam ball. During this pretraining session, mice were handled by an experimenter for 5–10 min and then allowed to move freely on the top of the ball that was rotated manually by the experimenter for another 5–10 min. The procedure was performed once a day and repeated for at least 5 d. The mice were then subjected to water restriction for at least 2 d before the start of behavioral training. Body weight and general appearance were examined daily to ensure that the mice exhibited no signs of abnormal behavior throughout the study. Mice were housed on a 12/12 h light/dark schedule (with lights on at 6 P.M. and off at 6 A.M. the next day). Experiments were performed during the dark phase of the cycle to enhance the locomotion of the mice.

The virtual unidirectional linear track was created using the editor function of OmegaSpace. Each subject was initially placed at the origin of the virtual linear track and started to run toward the other end after 1 s of waiting time (Fig. 1*B*a,*Bb*). The track segment was 120 cm long, measured as the number of rotations of the ball required to move from one end of the track to the other, multiplied by the circumference of the ball. The width of the track was set at 10 cm, although the mouse moved only one-dimensionally along the midline of the track with its view angle fixed toward the direction of movement. The track was divided into two segments and the walls of each segment were textured as follows: black circles on white background for 0–40 cm; white circles on black background for 40–120 cm. The floor was textured with black and white stripes aligned perpendicular to the direction of movement. The space above the track was gray. Three large objects (“tower,” “mountain,” and “building”) were placed outside the track so that the mouse running the track could use them as global cues. The target zone was defined typically at 60–80 cm from the origin and denoted with green panels on the walls and floor as local cues. Delivery of the water reward was triggered by different events in the nondelayed and delayed tasks (Fig. 1*C*). In the nondelayed task, three 5–µl drops of water were given at 1-s intervals whenever the mouse entered the target zone. In the delayed task, the mouse needed to stay within the target zone for a period of time to trigger the reward delivery. As in the nondelayed task, up to three water drops were given but delivery was stopped if the mouse moved out of the target zone. The mice were trained with an incrementally extended period of delay (1.0 and 1.5 s). As the mice usually ran through the target zone for <1.0 s if they moved without stopping, a delay of 1.5 s was sufficiently long and used in the experiments that examined the effects of cue manipulations, CNQX infusions, target shifts and Shank2 deletion. When the mice reached the far end of the track, they were automatically transferred to the origin after a 3 s wait to start a new trial.

Mice underwent a total of three to five weeks of behavioral training for the nondelayed and delayed tasks with two sessions per day. The training for the nondelayed task lasted for 4–11 sessions and the delayed task for 10–18 sessions (Fig. 1*D*). In the nondelayed phase of training, mice received no assistance from the experimenter, and data for all sessions were analyzed. In the following delayed phase, mice were assisted by the experimenter to stay in the target zone in the first session of each day throughout the training, whereas they were unassisted in the second session. For this reason, only the data for the unassisted sessions were analyzed. Assistance involved the experimenter holding the ball manually for the delay period when the mouse entered the target zone, and releasing it when the reward was delivered.

In each session, the mouse was head-fixed above the ball and was not anesthetized while being placed in the VR apparatus. Before starting a session, we waited for a few minutes until the head-fixed mouse became settled in the darkness. Each session was 10 min long, except in the cue manipulation experiments, when the session was 7.5 min long. Within an experimental day, recordings of the first and the second sessions of the subject groups usually started at 10 A.M. and 2 P.M., respectively, and intersession intervals were thus ∼4 h. The mice were returned to their home cage between sessions.

### Cue manipulation experiments

Mice trained for the delayed task were divided into two groups, each of which were subjected to experiments with mutually opposite orders of cue manipulation to control for a possible learning effect by repeated exposure to environments with limited cue availability. The experiments lasted for 3 d. On each day, the behavioral data with no cue manipulations were collected in the first sessions of the day and the data with manipulations in the second sessions. The session duration was shortened to 7.5 min to avoid the influence of reduced motivation for reward in the second sessions. A different set of visual cues were removed each day. For example, if the order of removal was “local-global-global plus local” for the first group of animals, this order was reversed in the other group. Some of the data were collected in experiments using a spherical screen for projection of the VR (*n* = 6 mice). These data were qualitatively similar to those obtained with a single LCD monitor (*n* = 5 mice) and the two datasets were therefore combined for statistical analysis.

### Intrahippocampal CNQX infusion

The water-soluble competitive AMPA/kainate-type glutamate receptor antagonist CNQX disodium salt (Tocris) was dissolved in cortex buffer (123 mM NaCl, 5 mM KCl, 10 mM glucose, 2 mM CaCl_2_, 2 mM MgCl_2_, and 10 mM HEPES, pH 7.4) at a concentration of 1 mg/ml (3.45 mM). The drug or vehicle was infused with a glass pipette connected to a 10-μl syringe via polyethylene tubing at a rate of 0.25 μl/min for 2 min (0.5 μl of total infusion volume). The coordinates were 1.8 mm posterior and 1.6 mm lateral from Bregma and 1.4 mm deep from the skull surface. After termination of infusion, the pipette was left undisturbed for an additional 2 min to avoid back flow. The infusion was performed bilaterally. After withdrawal of the pipette, the craniotomy was filled with a small amount of bone wax (Lukens, Surgical Specialties).

Preinfusion and postinfusion behavioral testing were performed one day before and 20–30 min after the infusion, respectively. Data for recovery from the CNQX infusion were collected 1–3 d after the infusion. After all experiments were conducted, mice were transcardially perfused with 4% paraformaldehyde in PBS. Infusion sites were then verified by making coronal sections and confirming that the trace of the pipette terminated in the dorsal CA1 region of the hippocampus.

### Target shift experiments

A separate set of mice were trained for the delayed task and divided into two groups. In the first group, mice initially trained with the original target location (target 1) were further trained to recognize a distally shifted (i.e., farther from the start position) new target location (target 2) for the following four sessions. This experiment was further followed by the second set of target shift experiments that contained a preshift session with the distal target location (target 2) and four additional sessions with the reward and local cue positions shifted together proximally (i.e., closer to the start position) to another new location (target 3). In the second group, the mice pretrained with target 1 were subjected to the two relearning tasks with the target shifts in a different order, where the reward and local cue positions were shifted from target 1 to target 3, and then to target 2. The positions of targets 1, 2, and 3 were 60–80, 90–110, and 30–50 cm from the origin of the track, respectively.

### Statistics

Data are expressed as the means ± SEMs unless stated otherwise. Statistical tests were performed using GraphPad Prism version 6.05 (GraphPad Software; Table 1). Exact p-values are shown unless *p* < 0.0001 (Table 1).

**Table 1:** Statistical table

		Data structure	Type of test	*p* value	Statistical value and degrees of freedom (for parametric test) or sample size (for nonparametric test)
a	Distance travelled	Normality not assumed	Wilcoxon matched-pairs signed rank test	0.0005 (nondelayed early vs nondelayed late) and 0.13 (delayed early vs nondelayed late)	W_(12)_ = 78 and 40
	Running speed	Normality not assumed	Wilcoxon matched-pairs signed rank test	0.0024 (nondelayed early vs nondelayed late) and 0.58 (delayed early vs nondelayed late)	W_(12)_ = 72 and 15
	Run period	Normality not assumed	Wilcoxon matched-pairs signed rank test	0.0005 (nondelayed early vs nondelayed late) and 0.42 (delayed early vs nondelayed late)	W_(12)_ = 78 and 22
	Target period	Normality not assumed	Wilcoxon matched-pairs signed rank test	0.0005 (delayed early vs delayed late)	W_(12)_ = 78
	Success rate	Normality not assumed	Wilcoxon matched-pairs signed rank test	0.0005 (delayed early vs delayed late)	W_(12)_ = 78
b	Distance travelled (Fig. 4*A2*,*B2*,*C2*)	Normality not assumed	Wilcoxon matched-pairs signed rank test	0.24 (control vs no global), 0.10 (control vs no local), and 0.76 (control vs no cues)	W_(11)_ = 28, −38, and 8
	Target period (Fig. 4*A3*,*B3*,*C3*)	Normality not assumed	Wilcoxon matched-pairs signed rank test	0.52 (control vs no global), 0.0049 (control vs no local), and 0.042 (control vs no cues)	W_(11)_ = 16, 60, and 46
	Success rate (Fig. 4*A4*,*B4*,*C4*)	Normality not assumed	Wilcoxon matched-pairs signed rank test	0.70 (control vs no global), 0.0068 (control vs no local), and 0.014 (control vs no cues)	W_(11)_ = −9, 58, and 47
c	Distance travelled (Fig. 5*B*)	Normality assumed	Two-way ANOVA with Holm–Sidak *post hoc* multiple comparisons test	0.87 (interaction), 0.75 (infusion type), and 0.50 (treatment period); 0.90 (pre vs CNQX), 0.90 (CNQX vs rec), 0.66 (pre vs veh), and 0.66 (veh vs rec)	Interaction, *F*_(2,40)_ = 0.14; infusion type, *F*_(1,20)_ = 0.11; treatment period, *F*_(2,40)_ = 0.71
	Target period (Fig. 5*C*)	Normality assumed	Two-way ANOVA with Holm–Sidak *post hoc* multiple comparisons test	<0.0001 (interaction), 0.040 (infusion type), and <0.0001 (treatment period); <0.0001 (pre vs CNQX), <0.0001 (CNQX vs rec), 0.90 (pre vs veh), and 0.85 (veh vs rec)	Interaction, *F*_(2,40)_ = 19.4; infusion type, *F*_(1,20)_ = 4.81; treatment period, *F*_(2,40)_ = 25.9
	Success rate (Fig. 5*D*)	Normality assumed	Two-way ANOVA with Holm–Sidak *post hoc* multiple comparisons test	<0.0001 (interaction), 0.019 (infusion type), and <0.0001 (treatment period); <0.0001 (pre vs CNQX), <0.0001 (CNQX vs rec), 0.60 (pre vs veh), and 0.48 (veh vs rec)	Interaction, *F*_(2,40)_ = 19.2; infusion type, *F*_(1,20)_ = 6.55; treatment period, *F*_(2,40)_ = 32.2
d	Target period, 1st group (Fig. 7*A*)	Normality not assumed	Wilcoxon matched-pairs signed rank test	0.031 (target 1, pre 1 vs shift 1), 0.031 (target 1, pre 2 vs shift 5), 0.031 (target 2, pre 1 vs shift 1), 0.031 (target 2, pre 2 vs shift 5), 0.031 (target 3, pre 2 vs shift 5), and 0.031 (target 3, shift 5 vs shift 6)	W_(6)_ = −21, 21, 21,−21, 21, and 21
	Target period, 2nd group (Fig. 7*B*)	Normality not assumed	Wilcoxon matched-pairs signed rank test	0.031 (target 1, pre1 vs shift 1), 0.031 (target 1, pre 2 vs shift 5), 0.031 (target 1, shift 5 vs shift 6), 0.031 (target 2, pre 2 vs shift 5), 0.031 (target 3, pre 1 vs shift 1), and 0.031 (target 3, pre 2 vs shift 5)	W_(6)_ = −21, 21, −21, 21, 21, and −21
e	Success rate (Fig. 7*D*)	Normality not assumed	Wilcoxon matched-pairs signed rank test	0.0049 (distal vs proximal)	W_(12)_ = 60
f	Distance travelled (Fig. 8*C*)	Normality assumed	Two-way ANOVA with Holm–Sidak *post hoc* multiple comparisons test	0.94 (interaction), 0.36 (genotype) and 0.024 (task stage); 0.81 (nondelayed early KO vs nondelayed early WT), 0.81 (nondelayed late KO vs nondelayed late WT), 0.81 (delayed early KO vs delayed early WT), and 0.81 (delayed late KO vs delayed late WT)	Interaction, *F*_(3,33)_ = 0.13; genotype, *F*_(1,11)_ = 0.92; task stage, *F*_(3,33)_ = 3.58
	Target period (Fig. 8*D*)	Normality assumed	Two-way ANOVA with Holm–Sidak *post hoc* multiple comparisons test	0.0026 (interaction), 0.23 (genotype), and 0.0003 (task stage); 0.91 (nondelayed early KO vs nondelayed early WT), 0.25 (nondelayed late KO vs nondelayed late WT), 0.25 (delayed early KO vs delayed early WT), and 0.011 (delayed late KO vs delayed late WT)	Interaction, *F*_(3,33)_ = 5.84; genotype, *F*_(1,11)_ = 1.63; task stage, *F*_(3,33)_ = 8.35
g	Target 3 period (Fig. 8*H*)	Normality not assumed	Mann-Whitney *U* test	*p* = 0.0023 (KO vs WT)	*U*_(6,7)_ = 1
	Relearning index (Fig. 8*I*)	Normality not assumed	Mann-Whitney *U* test	*p* = 0.022 (KO vs WT)	*U*_(6,7)_ = 5

## Results

### Task design and behavior

The virtual linear track used in this study was designed as shown in Figure 1*B* (see Materials and Methods for details). A mouse had its head fixed above a Styrofoam spherical treadmill (Fig. 1*A*) and was allowed to move in the virtual environment from one end of the linear track to seek a water reward given at a target location along the track. The mice first learned to run in this environment in a “nondelayed” task, in which they were rewarded immediately whenever they entered the target location (Fig. 1*C*). After the mice learned to run in the VR environment, they were trained to recognize the target location in the “delayed” task, in which they needed to stay at the target for a short period of time to receive a reward. The training started with several sessions of the nondelayed task followed by ∼10–20 sessions of the delayed task with the delay period extended from 1.0–1.5 s after a few initial sessions (see Materials and Methods for details; Fig. 1*D*).

**Figure 2.**
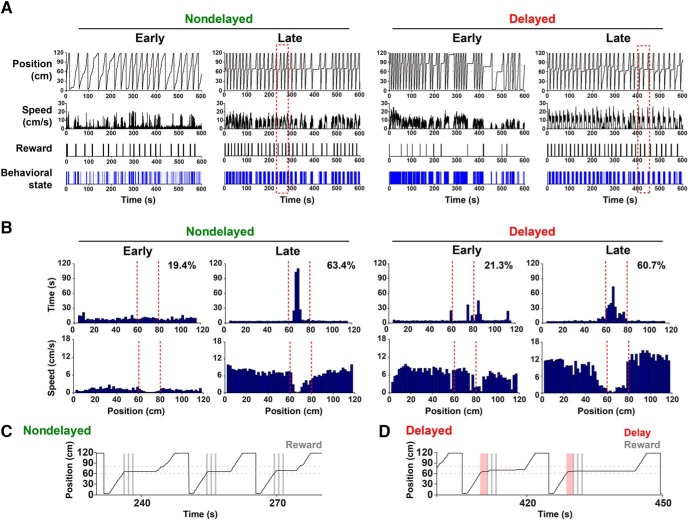
Behavioral changes induced by training. ***A***, Examples of behavioral changes induced by training in the nondelayed (left) and delayed tasks (right). The data shown are collected from either one of the early or late sessions (i.e., the first or last 2-3 sessions) in each version of the task and from the same mouse. The behavioral parameters presented are (from top to bottom) mouse position in the virtual linear track (position), running speed (speed), timing of reward delivery (reward), and behavioral state. Behavioral state indicates whether the mouse was running or immobile, and periods of running, during which the running speed of the mouse ≧2 cm/s, are indicated by blue vertical bars. ***B***, Histograms showing dwell time at each position on the track (upper panels) and average running speed across trials (lower panels) in early and late sessions of the nondelayed (left) and delayed tasks (right). The boundaries of the target zone are indicated by red dotted lines. Target periods are indicated in the upper right corner of the time histograms. ***C***, ***D***, Different sequences of behavior observed in the nondelayed (***C***) and delayed tasks (***D***). The areas of data enclosed by red dotted lines in ***A*** are shown at higher magnification. The shaded gray and red bars indicate the timing of reward delivery (reward) and waiting (delay), respectively.

**Figure 3.**
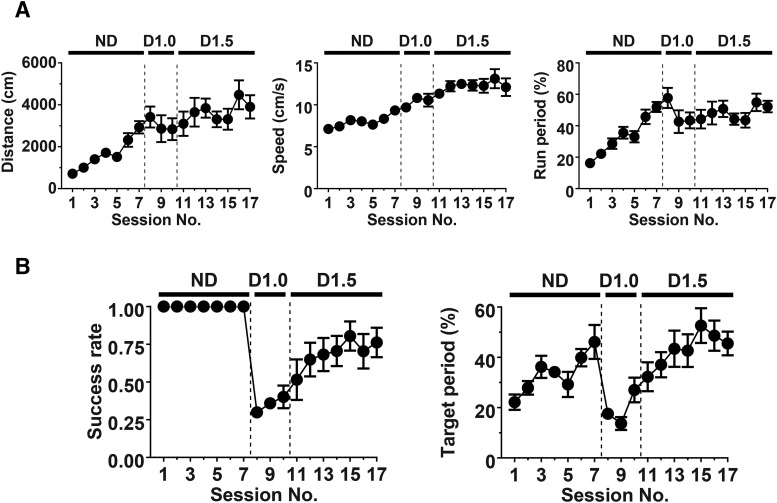
A summary time course of behavioral changes in mice trained in the nondelayed (ND) and delayed tasks. ***A***, The time course of changes in distance traveled (left), average running speed (middle), and the fraction of time spent for running (run period; right) during training in the ND and delayed tasks with 1.0- or 1.5-s delay (D1.0 and D1.5). The data shown were collected from training using a group of six mice. ***B***, Time course of changes in success rate (left) and target period (right) during training in the ND and delayed tasks with 1.0- or 1.5-s delay.

Training in the nondelayed task substantially enhanced the running behavior of mice in the virtual linear track (Fig. 2 for examples and Fig. 3 for a summary). The distance traveled, running speed and fraction of time that mice spent running (run period, which was calculated by thresholding running speed at 2 cm/s) significantly increased in the late stage of the nondelayed training compared with the early stage (distance traveled, early 1371 ± 200 cm vs late 3171 ± 414 cm, *p* < 0.001^a^; running speed, early 6.9 ± 0.2 cm/s vs late 9.8 ± 0.6 cm/s, *p* < 0.01^a^; run period, early 32.4 ± 4.6% vs late 51.3 ± 4.2%, *p* < 0.001^a^, n = 12 mice; Figs. 2*A*, 3*A*), demonstrating that mice effectively learn to run in the VR environment during this phase of training. Notably, during this training phase, mice frequently stopped when they passed the proximal border of the target zone, consumed the water reward triggered by the entry to the zone, and started to run again (Fig. 2*C*), resulting in a marked increase in the fraction of time spent at the target zone (target period; Figs. 2*B*, 3*B*).

When the task rule was switched to the delayed task, the fraction of successful trials (success rate), which was calculated as the number of rewarded trials divided by the total number of trials, decreased but eventually recovered after training (Figs. 2*A*, 3*B*). The virtual position and dwell time at each location of the track showed that the mice tended to stop occasionally inside or outside of the target zone in the early stage but began to stay specifically within the target zone in the late stage (Fig. 2*A*,*B*). The distance traveled, running speed and run period did not change considerably, compared with the late stage of the nondelayed task (distance traveled, delayed early 3755 ± 336 cm vs nondelayed late 3171 ± 414 cm, *p* > 0.05^a^; run period, delayed early 55.5 ± 4.0% vs nondelayed late 51.3 ± 4.2%, *p* > 0.05^a^; running speed, delayed early 10.2 ± 0.4 cm/s vs nondelayed late 9.8 ± 0.6 cm/s, *p* > 0.05^a^), suggesting that running behavior per se was not affected, but the distribution of time for running and staying within a session was altered by the change in the rules of the task. The target period and success rate substantially increased in the late stage of the delayed task training, compared with the early stage (Fig. 3*B*; target period, early 19.7 ± 2.1% vs late 47.2 ± 2.9%, *p* < 0.001^a^; success rate, early 0.34 ± 0.04 vs late 0.78 ± 0.06, *p* < 0.001^a^), suggesting that mice learned virtual goal localization through acquisition of the new task rule during this phase of training.

The dwell time histograms for the late stages of training in the nondelayed and delayed tasks appeared similar at first sight (Fig. 2*B*), but the behavioral sequences of the mice performing these tasks were qualitatively different. Mice trained with the delayed task stopped and stayed in the target zone first, then consumed the water reward triggered by the fact that they stayed in that zone, and then started to run again to exit the target zone (Fig. 2*D*). This “run-stay-rewarded-run” sequence was in contrast to the “run-rewarded-stay-run” sequence observed in the nondelayed task (Fig. 2*C*). Histograms of average running speed plotted against position clearly demonstrate that mice performing the delayed task ran at a relatively high constant speed from the start, slowed down near the proximal border of the target zone, stayed within the zone, and accelerated on exit from the target zone to the far end of the track (Fig. 2*B*). Furthermore, this alternation of running and staying, which was irregular in the early stage, became relatively regular in the late stage of training (Fig. 2*A*), suggesting that this characteristic pattern of behavior develops over the course of training.

### Effects of visual cue manipulations

Previous studies have demonstrated that visual cues guide the spatial navigation of rodents in virtual environments (Youngstrom and Strowbridge, 2012; Cushman et al., 2013). However, the relative importance of local (within-track) and global (out-of-track) cues remains to be clarified. We therefore removed each or all of these cues specifically from the track and assessed their contribution to the virtual spatial behavior of mice.

**Figure 4.**
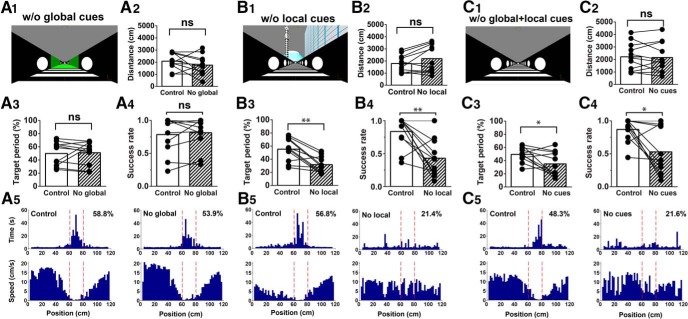
The effects of visual cue manipulations. ***A***, The effect of elimination of global cues. ***A1***, A camera view of the track without global cues. ***A2***, Distance traveled. ***A3***, Target period. ***A4***, Success rate. ***A5***, Histograms showing dwell time (upper panels) and running speed (lower panels) at each position on the track in control (left) and no-global-cue conditions (right). The boundaries of the target zone are indicated by red dotted lines. Target periods are indicated in the upper right corner of the time histograms. ***B***, ***C***, The effects of elimination of local cues (***B***) and both global and local cues (***C***). The same convention as A applies. **p < 0.01; *p < 0.05; ns, not significant, *n* = 11 mice.

Mice trained in the delayed task with a full set of visual cues were subsequently tested on the track without local and/or global cues. In the no-global-cue condition (Fig. 4*A1*), the distance traveled, target period and success rate did not change significantly compared with the control condition (Fig. 4*A2–A5*), suggesting that visual cues outside the track have little impact on guiding behavior. By contrast, the target period and success rate substantially decreased in the no-local-cue condition (Fig. 4*B1*, *B3–B5*; *p* < 0.01^b^, control vs no local), without significantly affecting distance traveled (Fig. 4*B2*; *p* > 0.05^b^, control vs no local). These results imply that visual cues located within the track at the target zone serve as landmarks to facilitate the goal localization of head-fixed mice in VR. Dwell time histograms indicate that these mice spent less time in the target zone (and more outside the target) in the no-local-cue-condition (Fig. 4*B5*). Finally, the no-global-plus-local-cue (no cues) condition significantly reduced the target period and success rate (*p* < 0.05^b^, control vs no cues) without altering distance traveled (*p* > 0.05^b^, control vs no cues; Fig. 4*C1–C5*). The extent of the reductions in the target period and success rate were comparable to those in the no-local-cue condition, suggesting that the effects of global plus local cue removal are primarily due to the effects of local cue removal. Interestingly, a subgroup of mice (e.g., 4 out of 11 mice in Fig. 4*C4*) performed the task equally well even without cues. These individuals may have used sensory modalities other than vision—most likely self-motion signals, to locate the target zone by path integration, potentially using the transition of different wall patterns before the target zone as a remaining within-track cue that was remote from the target zone (Fig. 1*B*).

### The dorsal hippocampus has an essential role in virtual cued goal localization

**Figure 5.**
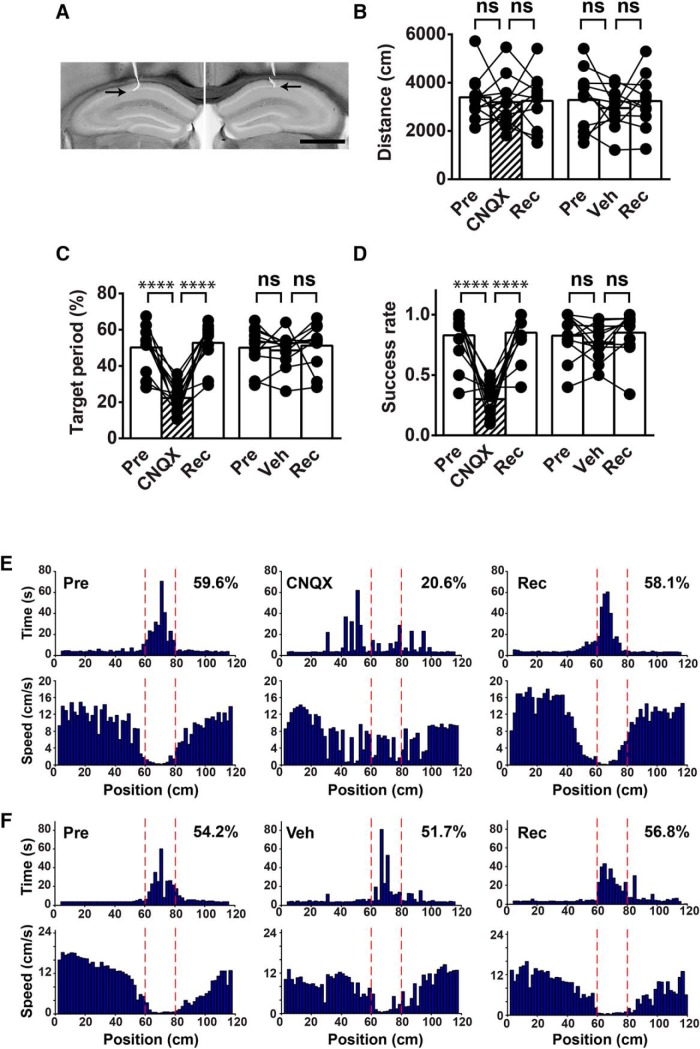
The effects of intrahippocampal CNQX infusion. ***A***, Bilateral infusion of CNQX into the dorsal CA1 region of the hippocampus. Typical traces of the infusion pipettes are shown by arrows in bright field micrographs of coronal sections. Images of each hemisphere shown here were taken from sections at slightly different anteroposterior axes due to experimental variability in pipette positioning and sectioning. Scale bar, 1 mm. ***B***, Distance traveled on the day before infusion (pre), 30 min after infusion of CNQX or vehicle (veh), and after 1-3 d of recovery from infusion (rec). ns, not significant. The same convention applies to ***C***, ***D***. ***C***, Target period. ****p < 0.0001, *n* = 12 mice. ***D***, Success rate. ***E***, ***F***, Histograms showing dwell time (upper panels) and running speed (lower panels) at each position on the track. Data for sessions on the day before infusion (pre, left), 30 min after infusion of CNQX (***E***; CNQX) or vehicle (***F***; veh, middle) and after 1-3 d of recovery from infusion (rec, right) are presented. The boundaries of the target zone are indicated by red dotted lines. Target periods are indicated on the upper right corner of the time histograms.

To examine whether the virtual cued goal localization described so far depends on hippocampal function, we infused the competitive AMPA/kainate-type glutamate receptor antagonist CNQX into the dorsal CA1 region of the hippocampus of mice trained in the delayed task (Fig. 5*A*). Specifically, we used the water-soluble CNQX disodium salt in our experiments to avoid potential tissue damage caused by organic solvents, such as dimethyl sulfoxide, commonly used to dissolve the water-insoluble, nonsalt form of CNQX. The pretrained mice exhibited good virtual goal localization performance on the day before infusion, as evaluated by the target period and success rate (Fig. 5*B–F*). After infusion, however, the CNQX-infused mice showed a significant reduction in these behavioral parameters (target period, infusion type × treatment period interaction: *F*_(2,40)_ = 19.4, *p* < 0.0001^c^, *post hoc* test for pre vs CNQX, *p* < 0.0001^c^; success rate, infusion type × treatment period interaction: *F*_(2,40)_ = 19.2, *p* < 0.0001^c^, *post hoc* test for pre vs CNQX, *p* < 0.0001^c^; Fig. 5*C*,*D*). Dwell time histograms demonstrate a tendency for CNQX-infused mice to spend more time outside the target zone (Fig. 5*E*). These defects were not observed in vehicle (veh)-infused control mice, precluding the possibility that the impaired virtual goal localization in the CNQX-infused mice was caused by the infusion procedure per se (Fig. 5*C*,*D*,*F*). The distance traveled was not significantly different before and after infusion in both CNQX- and veh-infused animals (infusion type × treatment period interaction: *F*_(2,40)_ = 0.14, *p* = 0.87^c^; *post hoc* test for pre vs CNQX, *p* = 0.90^c^; pre vs veh, *p* = 0.66^c^; Fig. 5*B*), implying that the suppression of excitatory synaptic transmission in the dorsal hippocampus specifically impairs virtual cued goal localization. Importantly, the effects of CNQX were reversible. The mice recognized the position of the target zone at a comparable extent to their preinfusion levels after 1–3 d of recovery (target period, *p* < 0.0001^c^, *post hoc* test for CNQX vs rec; success rate, *p* < 0.0001^c^, *post hoc* test for CNQX vs rec; Fig. 5*C*,*D*). These results suggest that transient inactivation of hippocampal excitatory synaptic activity impairs retrieval of previously acquired goal location memory but does not permanently abolish its stored memory traces.

### Rapid relearning of new goal locations in target shift experiments

**Figure 6.**
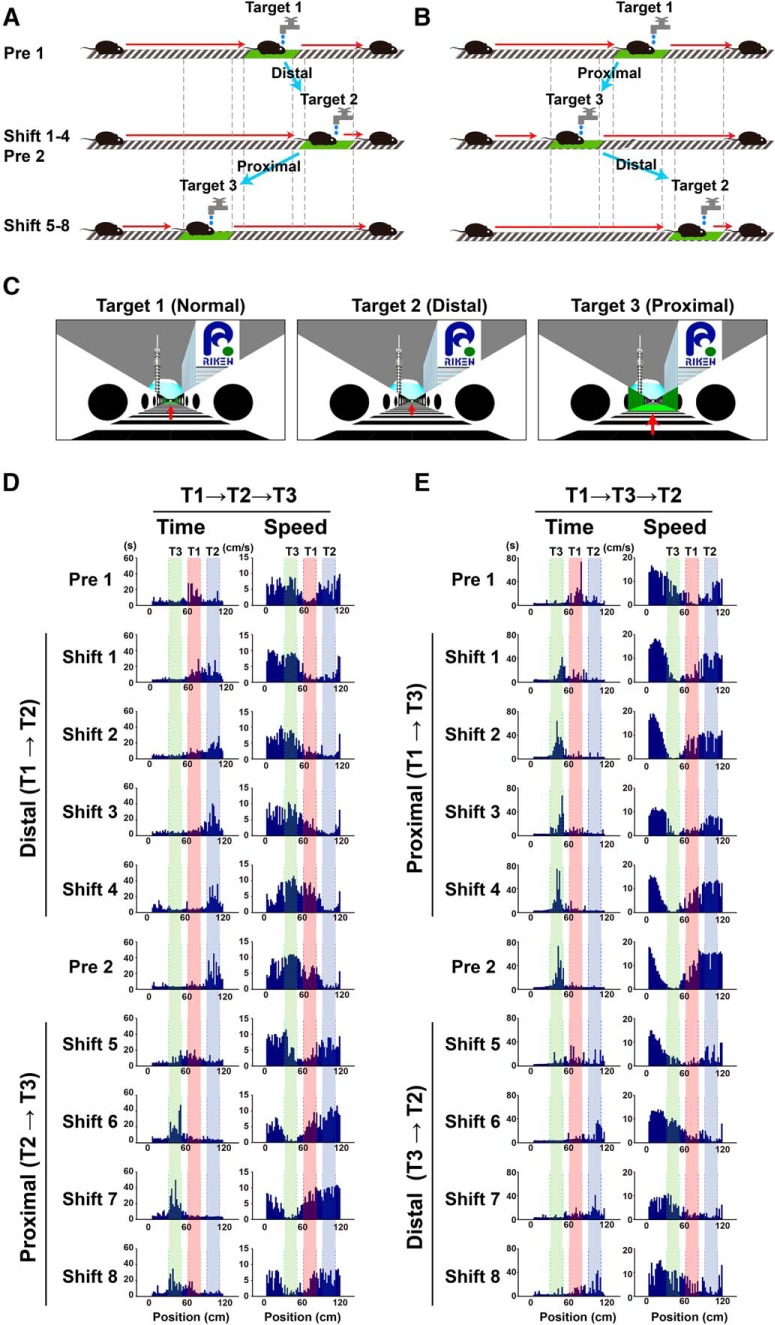
Rapid relearning of new target locations. ***A***, Mice were first trained to perform the delayed task for target 1 over a period of at least 10 sessions. Once training was complete (target 1 in pre 1 session), the reward position and local cues were moved distally to a new location, and mice were trained on the new location for the following four sessions (target 2 in shift 1–4 sessions). This experiment was further followed by a second round of target shift experiments, which consisted of one preshift session (target 2 in pre 2 session) and four additional training sessions with the reward position and local cues moved proximally to another new location (target 3 in shift 5–8 sessions). ***B***, In the second group, the experiments were conducted in a way similar to ***A*** but with the reverse order of shift directions. ***C***, Camera views for target 1 (normal, left), target 2 (distal, middle), and target 3 (proximal, right) from the same position of the linear track. Red arrows indicate the positions of the local cues at the target rendered in perspective. ***D***, Histograms showing a mouse’s dwell time at each position on the track (left) and running speed (right), displayed in chronological order of the whole experiment, from top to bottom. This animal was first trained for target 1 (T1, red), then target 2 (T2, blue), and finally target 3 (T3, green). ***E***, The same bidirectional behavioral plasticity seen in another mouse trained in the order of target 1, 3, and 2.

To gain further insight into the goal localization behavior of mice in VR, it was important to test whether mice could relearn new goal locations when the reward and local cues were relocated. Such relearning should require correct recognition of the local visual cues at new locations and reorganization of the previously learned cue–location associations. In our target shift paradigm, we tested two types of target shifts (Fig. 6*A–C*). In the first group, mice pretrained with a target position (target 1) were further trained to recognize a distally shifted new target position (target 2) for the following four sessions (distal shift; Fig. 6*A*). This experiment was subsequently followed by a second round of target shift experiments, which consisted of a preshift session (target 2) and four additional training sessions with the reward position and local cues shifted together proximally to another new location (target 3; proximal shift). In the second group, mice pretrained with the original target 1 were subjected to the two relearning tasks in reverse order (Fig. 6*B*). With this protocol, we could study the effects of shift directions separately from possible influences of the order of the shift.

**Figure 7.**
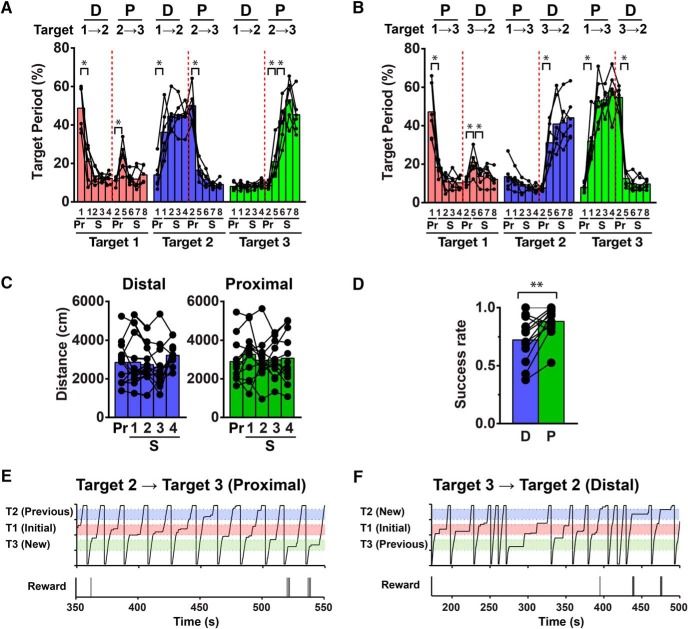
Summary data for target shift experiments and transient recall of old memories observed in the second target shift task. ***A***, Changes in the target periods at target 1 (red), 2 (blue), and 3 (green) over the target shift experiments depicted in Figure 6*A*. The target periods for each target were measured throughout all of the target shift experiments. The numbers on the *x*-axis indicate session numbers of pre (Pr) or shift (S) sessions, as shown in the example of Figure 6*D*. The first and second rounds of target shifts are divided by red vertical dotted lines. D, distal shift; P, proximal shift. *p < 0.05, *n* = 6 mice. The same convention applies to ***B***. ***B***, Changes in the target periods at target 1, 2, and 3 over all the target shift experiments depicted in Figure 6*B*. *n* = 6 mice. ***C***, Distance traveled represented separately for distal and proximal shifts. Data obtained from experiments depicted in Figure 6*A*,*B* were combined. *n* = 12 mice. ***D***, Average success rate of shift 2-4 and 6-8 sessions represented separately for distal and proximal shifts. Data obtained from experiments depicted in Figure 6*A*,*B* were combined. **p < 0.01, *n* = 12 mice. ***E***, A 200-s segment of data on mouse position (top) and timing of reward delivery (bottom) are shown. The mouse was first trained for target 1 (red), then target 2 (blue) and finally target 3 (green). In the first session after the target was moved from target 2 to target 3, the animal stopped in the original target zone target 1 a few times before it began to stop in the new target zone target 3. ***F***, The same behavior seen in a mouse trained in the order of target 1, 3, and 2.

In the first group, the fraction of time spent at target 1 rapidly and dramatically declined after the target zone was moved distally to target 2 (*p* < 0.05^d^, pre 1 vs shift 1; Figs. 6*D*, 7*A*). An increase in the fraction of time spent in target 2 was already apparent in the first postshift session (*p* < 0.05^d^, pre 1 vs shift 1) and reached near-saturation levels at the second postshift session. The peaks of dwell time and the troughs of average running speed plotted against position followed the move of the target zone in mice performing these tasks (Fig. 6*D*). Before the mice exhibited stable goal localization toward the new targets, they underwent intermediate behavioral states, particularly in sessions immediately after the target shift (i.e., the shift 1 session), where the distributions of the peak of dwell time and the width of the trough of running speed tended to be broader (Fig. 6*D*). The fraction of time spent in target 3 remained low during this relearning process.

In the second relearning training paradigm with the proximal target 3, the fraction of time spent in target 2 markedly decreased in the first postshift session (*p* < 0.05^d^, pre 2 vs shift 5; Fig. 7*A*). By contrast, the increase in the fraction of time spent in target 3 was moderate (*p* < 0.05^d^, pre 2 vs shift 5 and shift 5 vs shift 6) and reached a plateau level in the third session after the target shift. This result might be due to the following reason: when the target zone was shifted from target 2 to target 3, the fraction of time spent in target 1, where rewards were initially given but no rewards were delivered at that time, exhibited a small transient increase although the location was not denoted by local cues (*p* < 0.05^d^, pre 2 vs shift 5; Figs. 6*D*, 7*A*,*E*), suggesting the transient recall of old memories during relearning. The reappearance of halting at the initial target zone, invoked during a transition to the correct behavioral response from a distal to a proximal location, illustrates that the mice are not simply following the location of local cues. Instead, they remember the location at which they received the reward independently of local cues.

The animals in the second group exhibited essentially the same behavioral changes, except for the direction of these changes, as expected given the opposite order of target shift (Figs. 6*B*,*E*, 7*B*,*F*). The fraction of time spent in target 1 drastically declined in the first session after the target location was shifted proximally to target 3 (*p* < 0.05^d^, pre 1 vs shift 1). The increase in the fraction of time spent in target 3 was already clear in the first postshift session (*p* < 0.05^d^, pre 1 vs shift 1). In the second relearning training paradigm with the distal target 2, the fraction of time spent in target 2 and target 3 was markedly increased and decreased, respectively, in the first postshift session (*p* < 0.05^d^, pre 2 vs shift 5). In addition, a transient and significant increase in the fraction of time spent in target 1 after the second target shift was also observed (*p* < 0.05^d^, pre 2 vs shift 5 and shift 5 vs shift 6; Figs. 6*E*, 7*B*,*F*).

The distance that the mice ran during each session did not differ significantly throughout the experiments (Fig. 7*C*). Interestingly, although the behavioral plasticity induced by proximal and distal shifts is equally rapid and robust in quality, mice demonstrated a slight but significantly greater success rate in proximal shifts than in distal shifts (*p* < 0.01^e^, distal vs proximal; Fig. 7*D*), raising the possibility that mice may be able to perform better when the reward and local cues become available soon after the start of a new trial. Overall, these results demonstrate that head-fixed mice can relearn new virtual goal locations regardless of the order and direction of target shifts. This relearning occurs very rapidly compared with the initial period of training to locate the original target zone (Figs. 3*B*, 7*A*,*B*).

### Shank2 has an indispensable role in learning and relearning of virtual goal locations

Finally, to gain insights into the molecular mechanisms underlying learning of virtual goal localization, we tested mice lacking the postsynaptic scaffolding protein Shank2. This protein interacts with a number of synaptic proteins, including postsynaptic receptors, signaling molecules and cytoskeletal proteins, and acts as a hub of protein complexes at the postsynaptic density of excitatory synapses (Sheng and Kim, 2000; Hayashi et al., 2009). Shank2 is expressed abundantly in hippocampal pyramidal neurons (Böckers et al., 2004). In addition, mutations in the *Shank2* gene have been identified in individuals with autism spectrum disorders and mental retardation (Berkel et al., 2010). The Shank2-deficient mouse model, which mimics a microdeletion found in human patients, exhibits impaired plasticity at Schaffer collateral-CA1 pyramidal cell synapses and spatial learning in the Morris water maze task as well as autism-related behavioral phenotypes (Won et al., 2012), making this gene one of the most compelling mechanistic targets for this study.

**Figure 8.**
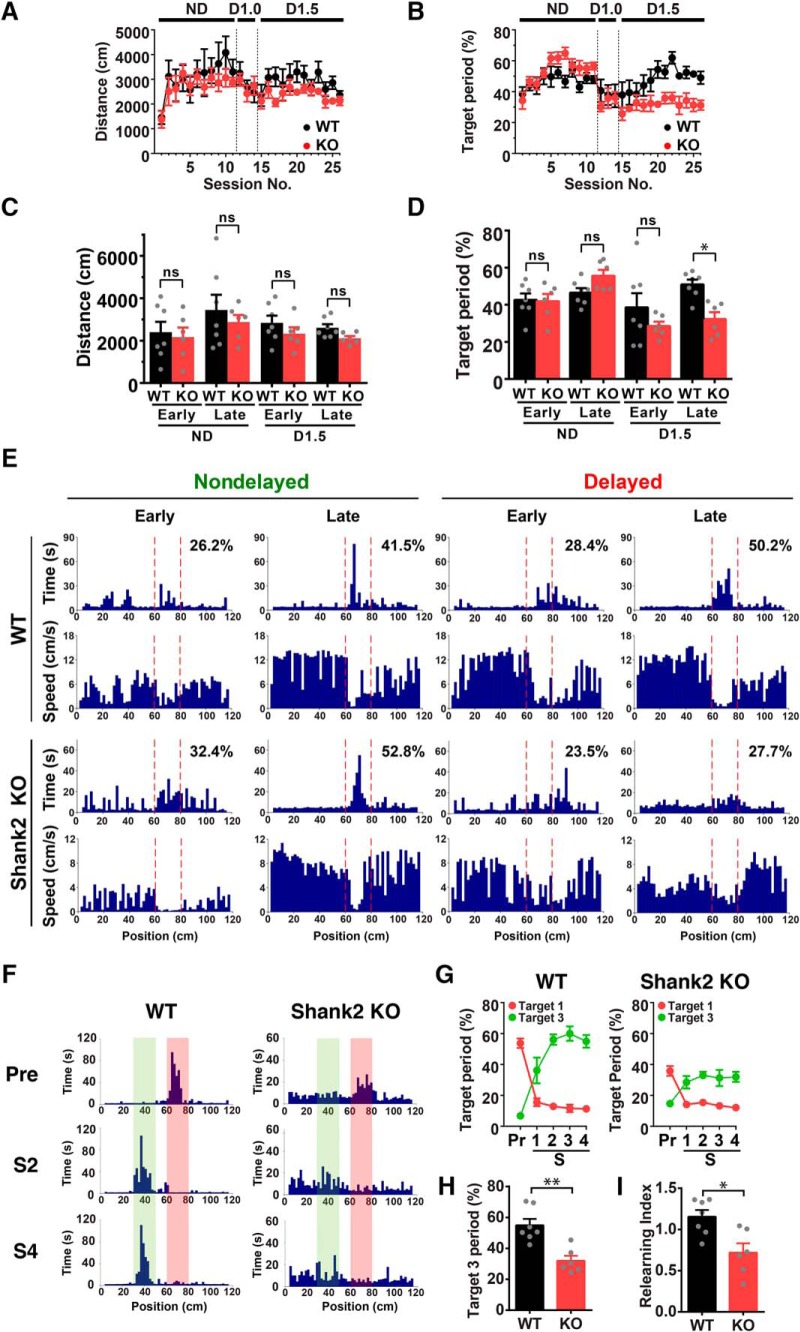
Impaired learning and relearning of virtual cued goal localization in Shank2-deficient mice. ***A***, ***B***, The time course of changes in distance traveled (***A***) and the target period (***B***) of wild-type (WT, black) and Shank2-deficient mice (KO, red) during training in the nondelayed (ND) and delayed tasks with 1.0- or 1.5-s delay (D1.0 and D1.5). ***C***, ***D***, Average distance traveled (***C***) and target periods (***D***) of wild-type (WT) and Shank2-deficient mice (KO) in the early and late phases of ND and D1.5. *p < 0.05; ns, not significant; *n* = 6–7 mice. ***E***, Histograms showing mouse dwell time and running speed at each position on the track in early and late sessions of the ND (left) and delayed tasks (right) for wild-type (top) and Shank2-deficient mice (bottom). Target periods are indicated in the upper right corner of the time histograms. ***F***, Dwell time histograms of wild-type and Shank2-deficient mice in pre, shift 2 (S2) and shift 4 (S4) sessions of the target shift experiments. The positions of target 1 and target 3 are indicated in red and green, respectively. ***G***, Changes in the target periods in target 1 (red) and target 3 (green) in the target shift experiments for wild-type (left) and Shank2-deficient mice (right). Pr, pre; S, shift. ***H***, Target periods of wild-type and Shank2-deficient mice for target 3 in shift 4 sessions. **p < 0.01, *n* = 6–7 mice. ***I***, Relearning indices of wild-type and Shank2-deficient mice, which were calculated as net changes in the target periods for target 3 between pre and shift 4 over those for target 1. *p < 0.05, *n* = 6-7 mice.

We trained littermate wild-type and homozygous Shank2-deficient mice in the nondelayed and delayed tasks (Fig. 8). The distance traveled and target period of Shank2-deficient mice were comparable to wild-type mice in the nondelayed and early delayed phases of training (Fig. 8*A–D*). However, Shank2-deficient mice lacked the learning-induced increase in dwell time in the target zone and spent significantly less time there in the late stage of training in the delayed task (task stage × genotype interaction: *F*_(3,33)_ = 5.84, *p* < 0.01^f^; *post hoc* test for KO vs WT in late D1.5, *p* < 0.05^f^; Fig. 8*B*,*D*,*E*), although the distance traveled was comparable to those of wild-type animals (task stage × genotype interaction: *F*_(3,33)_ = 0.13, *p* = 0.94^f^; *post hoc* test for KO vs WT in late D1.5, *p* = 0.81^f^; Fig. 8*A*,*C*). Furthermore, relearning after the target shift, as quantified by fractional increases in the dwell time in the new target over decreases in the old one, was also impaired in Shank2-deficient mice (target 3 period, *p* < 0.01^g^; relearning index, *p* < 0.05^g^; KO vs WT; Fig. 8*F–I*). Collectively, this evidence demonstrates that Shank2 plays an indispensable role in the learning and relearning of virtual goal localization.

## Discussion

We established a hippocampus-dependent cued goal location task for head-fixed mice in virtual environments. Unlike in real world situations, the modalities and quality of sensory information available to the subjects are limited in these environments (Youngstrom and Strowbridge, 2012; Ravassard et al., 2013). We therefore adopted a relatively simple task that involves only running and halting, but not turning behavior, to minimize the length of the training period. In this paradigm, head-fixed mice, motivated by the positive reinforcement of a water reward, were able to learn to visually locate and stay in the target zone, demonstrating that this paradigm requires the integrative use of different behavioral elements such as perception, recognition, decision and action. Furthermore, the target shift experiments, designed after the platform relocation test in the real-world Morris water maze task, can elicit rapid relearning of new goal locations in head-fixed mice. Importantly, our simplified VR setup using a single wide LCD monitor demonstrates that a toroidal screen to provide mice with a strong sense of immersion is not absolutely necessary (Hölscher et al., 2005; Youngstrom and Strowbridge, 2012). This simple set up will further facilitate the use of VR in standard laboratories as a tool to study the neural mechanisms underlying sensorimotor integration, spatial navigation and learning.

Among the many variations of spatial tasks performed in the real world, our VR paradigm is perhaps most similar to the annular water maze task used to study place recognition memory in rats (Hollup et al., 2001a; Brun et al., 2002; Fyhn et al., 2002). Both that task and ours are hippocampus-dependent and do not require complex two-dimensional navigational strategies after the subjects recognize the goal locations. One important difference, however, is that local visual cues presented at the goal play a dominant role in our task in head-fixed VR. As such cued tasks are generally considered hippocampus-independent in real-world situations (Morris et al., 1982; Hollup et al., 2001a; see also Diviney et al., 2013), this paradoxical finding could be because the relative influence and hippocampal dependence of global and local cues may differ when they are presented on a two-dimensional computer screen in head-fixed VR. The global cues presented in our unidirectional task may be too stationary to provide useful directional and distance information. Instead, salient local visual cues may take over this role (Youngstrom and Strowbridge, 2012; Chen et al., 2013) and help with visual scene recognition, potentially mediated by hippocampus-dependent visual memory (Prusky et al., 2004; Kim and Lee, 2012). Learning a visual discrimination task in VR elicits increasingly distinguishable representations of task-relevant stimuli and the emergence of anticipatory and behavioral choice-related signals in the mouse primary visual cortex (Poort et al., 2015). The sensory and nonsensory information shaped by learning at the level of visual information processing may inform the hippocampus to perform better goal localization. Local visual cues in VR could also be considered as object images presented at particular virtual locations. Their importance in virtual cued goal localization points to the possible involvement of a cognitive process related to object-place conjunction memory in the real world, rather than memory for place alone (Eichenbaum et al., 1999; Komorowski et al., 2009; Manns and Eichenbaum, 2009; Deshmukh and Knierim, 2013).

Transient and reversible suppression of virtual cued goal localization by CNQX infusion implies that information stored in the dorsal hippocampus is necessary for this behavior, consistent with past findings that fast excitatory synaptic transmission in the hippocampus is required for the retrieval of spatial memory in real environments (Riedel et al., 1999). In our task, the mice need to make a decision to halt at the target zone for the correct behavioral response, and to make this decision, they have to recognize whether they are approaching or have reached the remembered target zone. Theories and evidence for goal-directed navigation suggest that the hippocampus provides real-time information on the animal's current location to downstream neurons that represent its proximity to the goal (Burgess and O’Keefe 1996; Hok et al., 2005; Hirel et al., 2013; Erdem et al., 2015), as well as that the hippocampus itself represents information on learned goal locations (Hollup et al., 2001b; Hok et al., 2007; Dupret et al., 2010; McKenzie et al., 2013). Notably, it has been reported that sequences of place cell activity that encode future spatial trajectories to a remembered goal emerge in the hippocampus before goal-directed navigation (Pfeiffer and Foster, 2013). The inactivation of hippocampal activity in the present study may have prevented recall of the virtual goal location memory by transient suppression of the processes described above. The impaired learning and relearning of the delayed virtual goal location task in Shank2-deficient mice suggests that the integrity of postsynaptic protein complexes at glutamatergic synapses is required for encoding and updating of cued goal location memory in VR. Although Shank2 is deleted in the whole brain in these mice, their notable hippocampal phenotype and the prominent Shank2 expression in hippocampal pyramidal neurons of normal mice (Böckers et al., 2004; Won et al., 2012) suggest that the learning deficits observed in this study likely resulted from hippocampal deficits.

Our VR setup and behavioral paradigm for head-fixed mice can be readily combined with electrophysiology and imaging. In particular, our delayed task consists of different behaviors and perceptions, such as approaching the target zone, staying and waiting for the reward, exiting the target zone after reward consumption, and sudden transfer from the end of the track. Therefore, elucidating the distinct hippocampal neural circuit dynamics underlying each of these behavioral states as well as the plasticity of hippocampal network functions induced by learning and relearning will be of great interest in future physiologic studies. In regard to calcium imaging, the use of transgenic mice that express genetically encoded calcium indicators by various cell type- and region-specific targeting strategies will be helpful for efficient and reliable data collection as they do not require individual labeling of each trained animal (Chen et al., 2012; Zariwala et al., 2012; Sato et al., 2015). Finally, the experiments using Shank2-deficient mice clearly demonstrate that our VR paradigms are immediately applicable to studies of developmental and psychiatric disorders. Indeed, these disorders could have impaired development and function of neuronal circuits in their etiologies, as suggested by past functional imaging studies (Sato and Stryker, 2010; Gonçalves et al., 2013; Krishnan et al., 2015; Luongo et al., 2016). Our findings that Shank2-deficient mice exhibit impaired learning and relearning in the delayed tasks are reminiscent of intellectual disability and the behavioral inflexibility often seen in autistic patients and may thus provide a useful experimental framework for evaluating the efficacies of potential therapeutic agents for the treatment of this disorder (Spooren et al., 2012; Jiang and Ehlers, 2013).

## Synthesis

Reviewing Editor: Tatyana Sharpee, The Salk Institute for Biological Studies Decisions are customarily a result of the Reviewing Editor and the peer reviewers coming together and discussing their recommendations until a consensus is reached. When revisions are invited, a fact-based synthesis statement explaining their decision and outlining what is needed to prepare a revision will be listed below. The following reviewer(s) agreed to reveal their identity: Marianne Fyhn

The concerns raised by the expert reviewers were that (i) the task is closer to being a cued goal-location task than a place recognition task, and (ii) that the way the authors use terms “local” and “global” cues might not agree with established terminology. Regarding the last point, according to the expert reviewer, in real life 1-D track experiments, landmarks on the walls of a room would be considered to be global cues, but cues on the track itself are considered to be local cues.

Specific additional concerns by reviewers were as follows:

Reviewer 1:

Please cite and discuss https://www.ncbi.nlm.nih.gov/pmc/articles/PMC4503798/

Please rewrite the sentence: “Because such cued tasks are generally considered hippocampus-independent in real-world situations (Morris et al., 1982; Hollup et al., 2001a; see also Diviney et al., 2013), this paradoxical finding could be because the relative importance of, and hippocampal dependence of, global and local cues may differ when they are presented in a limited perspective on a two-dimensional computer screen placed distally in front of a head-fixed mouse”

Reviewer 2:

With regard to Figure 5B-D, the authors conclude that CNQX had a stronger effect on target period and success rate than vehicle. However, it seems as though they did not include CNQX and vehicle data in the same analysis but instead performed a series of pairwise comparisons. To substantiate this conclusion, the authors should have seen an interaction between infusion type (i.e., CNQX vs. vehicle) and treatment period (i.e., pre, drug, recovery), with subsequent tests showing that CNQX measures were decreased compared to pre and recovery and that vehicle measures were not.

Related to the above point, an analogous issue exists for the WT and KO data in Figure 8C-D. Instead of performing a series of separate pairwise comparisons, the authors should first test for an interaction between task stage (i.e., early vs. late) and genotype.

Additional major comments:

1. The description of animals that were used is unclear on page 5, lines 23-27. First, the authors write that “Male C57BL/6J mice and male and female Shank2-deficient mice were used”. But then they write, “In experiments using Shank2-deficient mice, data were compared between groups of homozygous mutant and wild-type littermate mice (3 males and 3 females for mutant and 3 males and 4 females for wild-type mice)”. Which is correct?

2. The authors should report the value of statistics and degrees of freedom, not just p-values.

3. With regard to Figure 5B-D, the authors conclude that CNQX had a stronger effect on target period and success rate than vehicle. However, it seems as though they did not include CNQX and vehicle data in the same analysis but instead performed a series of pairwise comparisons. To substantiate this conclusion, the authors should have seen an interaction between infusion type (i.e., CNQX vs. vehicle) and treatment period (i.e., pre, drug, recovery), with subsequent tests showing that CNQX measures were decreased compared to pre and recovery and that vehicle measures were not.

4. Related to the above point, an analogous issue exists for the WT and KO data in Figure 8C-D. Instead of performing a series of separate pairwise comparisons, the authors should first test for an interaction between task stage (i.e., early vs. late) and genotype.

5. I did not really understand the rationale for the relearning experiments. On page 14, at approximately line 13, I suggest that the authors explain better why it was important to show that animals could relearn different reward locations.

6. “Behavioral state” in Figure 2A is not well described. I presume it indicates running vs. immobility, but if so this should be stated clearly, and it should be clear to readers how exactly running and immobility were defined.

7. What do the shaded gray and red bars indicate in Figure 2C-D?

8. In Figure 7A-B, why are there 2 pre and 2 shifts for each target? Weren't the proximal and distal shifts only performed once for each group? I had a difficult time understanding this figure. Part of the issue is that the x-axes were not clearly explained in the legend. For example, what do the numbers in the x-axes indicate?

Minor comments:

1. On page 3, lines 21–24, references are missing after the sentence that begins with “These different environments have been used to study intracellular dynamics, local circuit mechanisms, cellular maps and dendritic activity of place cells…” I suggest including relevant citations after each of these examples.

2. On page 7, line 16, the authors write, “used in most experiments”. Is it possible to be more precise regarding the methods?

3. On page 7, line 20, the authors write, “Typically, the training for the non-delayed task lasted for 4-11 sessions…” Again, this wording is vague. Were there times when training did not last for 4–11 sessions and, if so, wouldn't it be better to state the exact range of training sessions in the experiments?

4. On page 7, lines 23–24, the authors write, “mice were assisted by the experimenter”. How exactly?

5. On page 8, line 5, the authors write, “intervals were typically 4 h”. Again, this wording is vague. Is it possible to be more specific than “typically”?

6. On page 9, lines 21–24, the description of the target shifts is not written in an especially clear manner. That is, in the passage that reads “and 4 additional sessions with the reward and local cue positions shifted proximally (i.e., closer to the start position) to another new location (Target 3).” In this passage, it is unclear whether Target 3 refers to the reward location or the local cue positions. Also, the subsequent sentence states, “In the second group, the mice trained with Target 1 were subjected to the two relearning tasks in reverse order”. What does this mean? Weren't all mice trained with Target 1?

7. On page 11, lines 22–23, the authors write, “suggesting that learning of virtual place recognition occurred during this phase of training”. Couldn't it also suggest that the animals learned the new task rule during this phase of training?

8. On page 13, line 11, the authors write, “using the transition of different wall patterns before the target zone as a remote cue”. Why would wall patterns be a remote cue? What do the authors mean by “remote cue”, and how exactly does it differ from “global cues” and “local cues” as defined in Figure 1B?

9. On page 20, lines 11–12, it is strange to cite these past studies after the phrase “future physiological studies”.

10. In the Figure 6C legend, the authors write, “Red arrows indicate the positions of the local cues rendered in perspective”. This means the local cues associated with the target, correct? That is, the green cues at the target site are the only local cues, correct? Perhaps this would be clearer if the authors added “at the target” after “positions of the local cues”.
